# A new IRMA module for analyzing whole-genome sequences from human metapneumovirus

**DOI:** 10.1128/mra.00901-25

**Published:** 2025-10-21

**Authors:** Emily E. Bendall, Adam S. Lauring

**Affiliations:** 1 Department of Microbiology and Immunology, University of Michigan 1259, Ann Arbor, Michigan, USA; 2 Division of Infectious Diseases, Department of Internal Medicine, University of Michigan 1259, Ann Arbor, Michigan, USA; DOE Joint Genome Institute, Berkeley, California, USA

**Keywords:** human metapneumovirus, HMPV, genome analysis, iterative refinement meta-assembler, consensus sequence, genomic surveillance

## Abstract

The large amount of genetic diversity in human metapneumovirus makes reference-based alignments difficult. We created a new module for the Iterative Refinement Meta-Assembler (IRMA) that performs alignment and consensus sequence generation without requiring subtyping and can handle duplications in the glycoprotein. This module increases the feasibility of genomic surveillance.

## ANNOUNCEMENT

Human metapneumovirus (HMPV) causes a significant number of respiratory infections each year, especially in young children (1). HMPV is genetically diverse with two antigenically distinct lineages (A and B) that cocirculate (2, 3). These two lineages have each split into two sublineages (A1, A2, B1, and B2), and A2 has further split into A2.1 and A2.2 (4). Most of the genetic diversity among subtypes is in G, the glycoprotein (5, 6). G is also highly variable within subtypes, with strains containing either a 111 or 180 nucleotide duplication currently circulating within A2.2 (7, 8).

There has been limited whole-genome sequencing of HMPV, despite potential public health benefits of genomic surveillance. One of the barriers to whole-genome sequencing is efficiently analyzing the sequence data due to the large amount of genetic diversity. Current library preparation methods do not require subtyping (9–11). However, the genetic diversity of HMPV hinders the ability to use a single reference to accurately assemble genomes for all samples.

To address this problem, we have developed an HMPV IRMA module. IRMA was developed for assembling highly variable RNA viruses (12). IRMA is reference-based, but it iteratively gathers reads and edits the reference genome, minimizing the effects of distance from the initial reference. It also allows for a different reference genome for each subtype, making prior subtyping unnecessary. To create the reference, we downloaded all whole genomes available on GenBank (accessed Oct. 18, 2024, “Metapneumovirus hominis”). Sequences were aligned using MAFFT v7 (13), and IQ-TREE 2 (14) was used to create a phylogeny. We used previously typed samples and the phylogeny to assign samples to A1, A2, A2.1, A2.2, A2.2 +111 nt duplication, A2.2 +180 nt duplication, B1, or B2 (Table 1). For each sublineage, we created a plurality consensus sequence using EMBL consensus generator (15) and a hidden Markov model using IRMA.

**TABLE 1:** The number of genomes used to create the consensus reference sequences^*a*^

Lineage	Number of genomes
A1	15
A2.1	47
A2.2	140
A2.2 (111)	103
A2.2 (180)	6
B1	92
B2	118
**Total**	**521**

^*a*^ Number in parentheses is the size of the G duplication.

To test the IRMA pipeline, we sequenced 181 samples from the Investigating Respiratory Viruses in the Acutely Ill (IVY) study (November 2024–April 2025) (16, 17) and from the Household Influenza Vaccine Effectiveness (HIVE ) study (2011–2022) (18). Nasal swabs were sequenced using the Respiratory Virus Oligos Panel v2 on an Illumina NextSeq 2000 (2 × 300, P1 chemistry).

**Fig 1:**
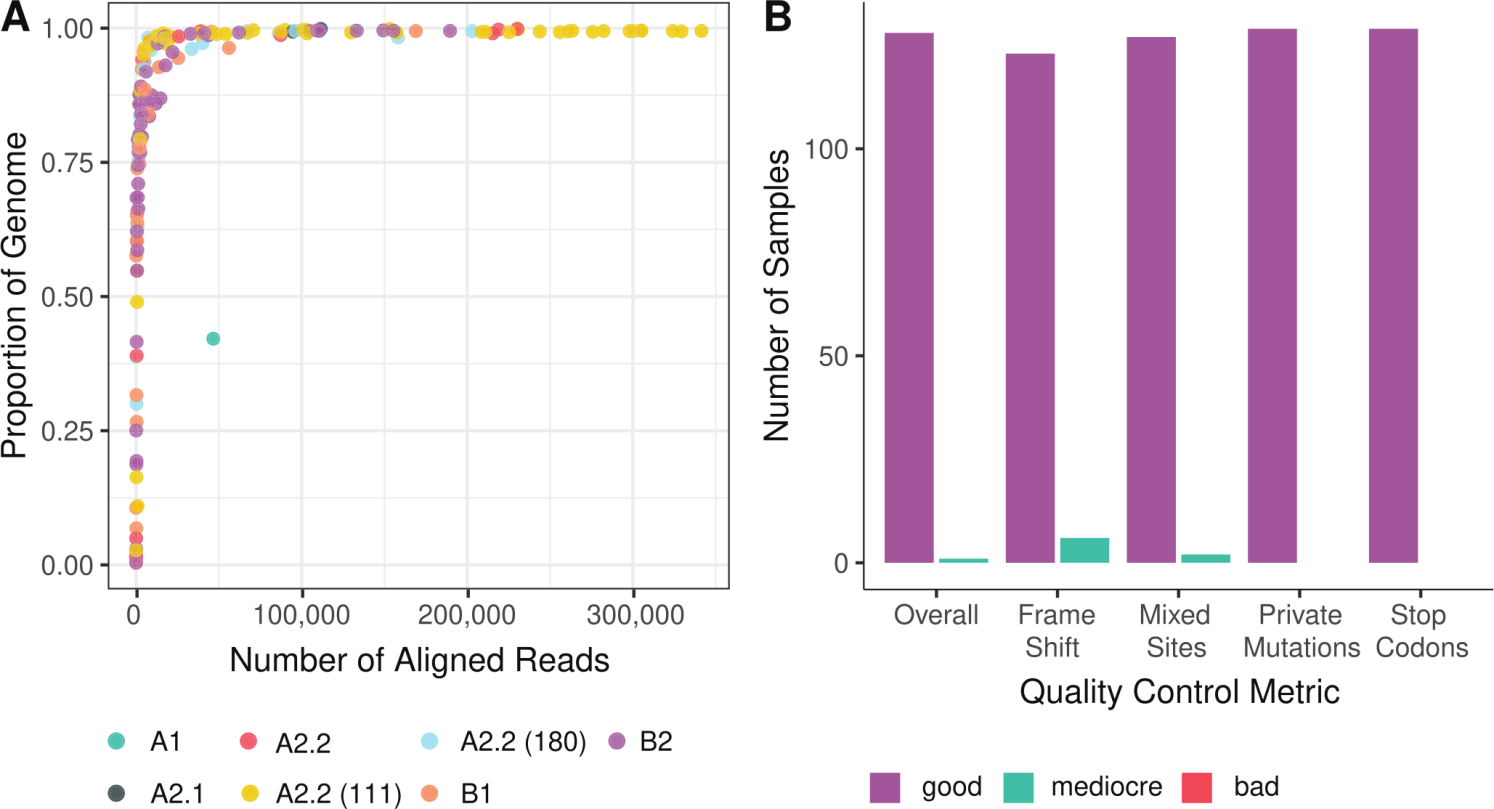
The IRMA module creates complete and high-quality HMPV consensus genomes. (**A**) Genome completeness. The number of reads aligned to the final reference versus the proportion of nucleotides present in the consensus genome. The color is the reference genome used for each sample. (**B**) Quality control metrics from Nextclade for samples with at least 75% coverage of the genome.

The consensus sequences generated by IRMA were complete or nearly complete genomes (Fig. 1A). A2.1, A2.2, B1, and B2 lineages were present (19). Lineages were consistent with previous qPCR subtyping (A or B) (20). We were able to detect the 111-nt (42 samples) and 180-nt (11 samples) insertions in a subset of A2.2 samples, showing that the IRMA module can handle samples with or without a duplication. No systematic issues were detected in the alignments (Fig. 1B). The IRMA module is suitable for Illumina and Nanopore sequencing. For Nanopore sequencing, the config file would need to be altered (see Flu module in IRMA for example). For Illumina sequencing, read lengths shorter than 300 bp compromise accurate detection of duplications.

## Data Availability

The module and consensus sequences are available at https://github.com/lauringlab/HMPV_IRMA_module. To use, place the file inside the modules folder of IRMA and follow the instructions at https://wonder.cdc.gov/amd/flu/irma/index.html. Sequences are available at BioProject PRJNA1304962.

## References

[1] Akingbola A, Adegbesan A, TundeAlao S, Adewole O, Ayikoru C, Benson AE, Shekoni M, Chuku J. 2025. Human metapneumovirus: an emerging respiratory pathogen and the urgent need for improved diagnostics, surveillance, and vaccine development. Infect Dis (Lond) 57:304–310. doi:10.1080/23744235.2025.2453824 39862211

[2] van den Hoogen BG, Herfst S, Sprong L, Cane PA, Forleo-Neto E, de Swart RL, Osterhaus ADME, Fouchier RAM. 2004. Antigenic and genetic variability of human metapneumoviruses. Emerg Infect Dis 10:658–666. doi:10.3201/eid1004.030393 15200856 PMC3323073

[3] Boivin G, Mackay I, Sloots TP, Madhi S, Freymuth F, Wolf D, Shemer-Avni Y, Ludewick H, Gray GC, LeBlanc E. 2004. Global genetic diversity of human metapneumovirus fusion gene. Emerg Infect Dis 10:1154–1157. doi:10.3201/eid1006.031097 15207075 PMC3323143

[4] Huck B, Scharf G, Neumann-Haefelin D, Puppe W, Weigl J, Falcone V. 2006. Novel human metapneumovirus sublineage. Emerg Infect Dis 12:147–150. doi:10.3201/eid1201.050772 16494734 PMC3291390

[5] Papenburg J, Carbonneau J, Isabel S, Bergeron MG, Williams JV, De Serres G, Hamelin M-È, Boivin G. 2013. Genetic diversity and molecular evolution of the major human metapneumovirus surface glycoproteins over a decade. J Clin Virol 58:541–547. doi:10.1016/j.jcv.2013.08.029 24041471

[6] Ishiguro N, Ebihara T, Endo R, Ma X, Kikuta H, Ishiko H, Kobayashi K. 2004. High genetic diversity of the attachment (G) protein of human metapneumovirus. J Clin Microbiol 42:3406–3414. doi:10.1128/JCM.42.8.3406-3414.2004 15297475 PMC497604

[7] Piñana M, Vila J, Gimferrer L, Valls M, Andrés C, Codina MG, Ramón J, Martín MC, Fuentes F, Saiz R, Alcubilla P, Rodrigo C, Pumarola T, Antón A. 2017. Novel human metapneumovirus with a 180-nucleotide duplication in the G gene. Future Microbiol 12:565–571. doi:10.2217/fmb-2016-0211 28604069

[8] Saikusa M, Nao N, Kawakami C, Usuku S, Sasao T, Toyozawa T, Takeda M, Okubo I. 2017. A novel 111-nucleotide duplication in the G gene of human metapneumovirus. Microbiol Immunol 61:507–512. doi:10.1111/1348-0421.12543 28960538

[9] Illumina. 2020. Detection and characterization of respiratory viruses, including SARS-CoV-2, using illumina RNA prep with enrichment. 1270-2020-002-A

[10] Groen K, van Nieuwkoop S, Bestebroer TM, Fraaij PL, Fouchier RAM, van den Hoogen BG. 2021. Whole genome sequencing of human metapneumoviruses from clinical specimens using MinION nanopore technology. Virus Res 302:198490. doi:10.1016/j.virusres.2021.198490 34146613

[11] Tulloch RL, Kok J, Carter I, Dwyer DE, Eden J-S. 2021. An amplicon-based approach for the whole-genome sequencing of human metapneumovirus. Viruses 13:499. doi:10.3390/v13030499 33803613 PMC8003040

[12] Shepard SS, Meno S, Bahl J, Wilson MM, Barnes J, Neuhaus E. 2016. Viral deep sequencing needs an adaptive approach: IRMA, the iterative refinement meta-assembler. BMC Genomics 17:708. doi:10.1186/s12864-016-3030-6 27595578 PMC5011931

[13] Katoh K, Standley DM. 2013. MAFFT multiple sequence alignment software version 7: improvements in performance and usability. Mol Biol Evol 30:772–780. doi:10.1093/molbev/mst010 23329690 PMC3603318

[14] Minh BQ, Schmidt HA, Chernomor O, Schrempf D, Woodhams MD, von Haeseler A, Lanfear R. 2020. IQ-TREE 2: new models and efficient methods for phylogenetic inference in the genomic era. Mol Biol Evol 37:1530–1534. doi:10.1093/molbev/msaa015 32011700 PMC7182206

[15] Madeira F, Madhusoodanan N, Lee J, Eusebi A, Niewielska A, Tivey ARN, Lopez R, Butcher S. 2024. The EMBL-EBI job dispatcher sequence analysis tools framework in 2024. Nucleic Acids Res 52:W521–W525. doi:10.1093/nar/gkae241 38597606 PMC11223882

[16] Surie D, Yuengling KA, DeCuir J, Zhu Y, Lauring AS, Gaglani M, Ghamande S, Peltan ID, Brown SM, Ginde AA, et al.. 2024. Severity of respiratory syncytial virus vs COVID-19 and influenza among hospitalized US adults. JAMA Netw Open 7:e244954. doi:10.1001/jamanetworkopen.2024.4954 38573635 PMC11192181

[17] US. Centers for Diesease Control and Prevention. 2024. Investigating respiratory viruses in the acutely Ill (IVY) network. Available from: https://www.cdc.gov/flu-vaccines-work/php/vaccine-effectiveness/ivy.html

[18] Bassiouni SS, Foster-Tucker JE, Callear AP, Godonou E-T, Smith M, Johnson E, Martin ET, Monto AS. 2025. A comparative profile of the burden of human metapneumovirus, respiratory syncytial virus, and influenza in the HIVE cohort, 2010-2022. J Infect Dis 232:S101–S108. doi:10.1093/infdis/jiaf113 40668094 PMC12265056

[19] Aksamentov I, Roemer C, Hodcroft EB, Neher RA. 2021. Nextclade: clade assignment, mutation calling and quality control for viral genomes. JOSS 6:3773. doi:10.21105/joss.03773

[20] Sugimoto S, Kawase M, Suwa R, Kakizaki M, Kume Y, Chishiki M, Ono T, Okabe H, Norito S, Hosoya M, Hashimoto K, Shirato K. 2023. Development of a duplex real-time RT-PCR assay for the detection and identification of two subgroups of human metapneumovirus in a single tube. J Virol Methods 322:114812. doi:10.1016/j.jviromet.2023.114812 37741464

